# Preliminary Study of a Single Instillation of Low-Concentration High-Volume Silver Nitrate Solution for Chyluria: Is >10 mL Instillation an Absolute Contraindication in the Real World?

**DOI:** 10.3390/tropicalmed4040128

**Published:** 2019-10-16

**Authors:** Kensuke Mitsunari, Yushi Imasato, Toshifumi Tsurusaki

**Affiliations:** Department of Urology, The Japanese Red Cross Nagasaki Genbaku Hospital, 3-15 Mori-machi, Nagasaki 852-8511, Japan; imasato2001@gmail.com (Y.I.); ttsuru@bronze.ocn.ne.jp (T.T.)

**Keywords:** silver nitrate, chyluria, low concentration, high volume, single instillation

## Abstract

Silver nitrate instillation (SNI) is one form of treatment for chyluria. However, there is the opinion that a high volume of SNI (>10 mL) should be avoided because life-threatening complications have been reported. However, we have noticed that most severe complications occur in high-concentration treatments (≥1%), even with a small volume. In addition, a small volume (≤10 mL) of low-concentration (<1%) SNI occasionally causes recurrence. Based on these facts, we aimed to evaluate a preliminary study of a novel single-injection regimen of low-concentration high-volume SNI. In this preliminary study, nine patients who underwent SNI were retrospectively examined. Patient characteristics, anesthesia, procedure, efficacy, complications, and duration of hospital stay were investigated. The volume of silver nitrate solution was decided based on symptoms, findings of pyelography, and vital signs, and it was given as a single instillation. This study was approved by the Institutional Review Board. The ranges of silver nitrate concentration and volume were 0.1%–0.5% and 15–30 mL, respectively. In all patients, proteinuria and cloudy urine disappeared immediately. However, two patients had recurrence after the initial SNI. These two patients were subsequently treated with increasing concentrations of silver nitrate (0.2% and 0.5%) from 0.1%, and they had complete symptomatic relief without recurrence for more than 6 years. None of the patients experienced severe complications. In conclusion, our preliminary study showed that a single instillation of low-concentration (0.1%–0.5%) and high-volume (15–30 mL) SNI is safe and useful. It is worth considering as a treatment option for chyluria.

## 1. Introduction

Chyluria is caused by the communication between the lymphatic and pyelocaliceal systems, usually resulting from lymphatic rupture or fistulous connection into the urinary tract due to obstructive lymphatic stasis [1]. Chyluria can be classified as either parasitic-related or non-parasitic-related chyluria, and the most common parasitic cause is filariasis caused by *Wuchereria bancrofti* [2,3]. This parasite is spread by different types of mosquitoes, and these mosquitoes propagate in water-logged areas in high-temperature climates. Therefore, at present, chyluria is commonly seen in tropical and subtropical regions where filariasis is endemic [4]. However, we should note that global warming and the increasing number of travelers has led to the high prevalence of various mosquito-transmitted parasites [5,6]. A recent concern is the increasing proportion of patients with filarial chyluria in Europe, North America, as well as tropical countries. In addition, high temperatures, humidity, and expanding water-logged areas may contribute to the increasing number of patients in Southeast Asia, Australia, and Africa. 

Chyluria presents as cloudy urine, urinary tract infections, voiding disturbance, acute urinary retention due to chylous or hematochylous clots, and systemic edema due to protein loss [3]. In general, conservative treatment, such as bed rest, wearing a tight corset, a low-fat and high-protein diet, or diets containing medium-chain triglycerides, is indicated in early and mild cases [1]. On the contrary, sclerotherapy is recognized as the standard therapy for patients for whom conservative therapy is unsuccessful, and silver nitrate, povidone-iodine, sodium iodide, potassium bromide, and dextrose, or a combination of these agents, are used as sclerosants [1]. Among these agents, silver nitrate had been commonly used and well studied. Previous reports showed that silver nitrate instillation (SNI) is a relatively safe procedure with high success rates [7,8]. However, unfortunately, some serious and life-threatening complications have been reported [9,10,11]. Therefore, at present, povidone-iodine-based therapy is most commonly used because it has shown good results without lethal adverse events [1,12]. In contrast, there is not much information on the efficacy and safety of other agents as compared with silver nitrate and povidone-iodine. From these facts, there is a general agreement that povidone-iodine is the standard agent for sclerotherapy in patients with chyluria. 

As mentioned above, povidone-iodine and povidone-iodine-based therapies, such as a single use of 0.2% povidone-iodine, a combination of 5% povidone-iodine and 50% dextrose instillation, and 0.2% povidone-iodine and 76% urographin, have a high cure rate of over 90% immediately after therapy [12,13,14,15]. Unfortunately, recurrence rates and disease-free periods after such therapies are quite short; for example, 7 of 41 (17.1%) patients recurred, with a mean period of 18 months, in a regimen composed of 0.2% povidone-iodine, and 14.8% recurred after 9.25 weeks [13,14]. For recurrent chyluria, the same protocol was performed in almost all studies, and operations such as endoscopic treatment, nephrolysis, microsurgical procedures (lymphangiovenous and lymph-node–saphenous-vein anastomosis), renal autotransplantation, and nephrectomy may be indicated for patients with refractory chyluria and repeated failed therapy [3,12,14,15,16]. Thus, in addition to povidone-iodine, other effective and safe protocols are necessary to avoid surgical treatment in these patients. Although great care is required when SNI is used, we endeavored to focus again on silver nitrate based on the following reasons: (1) There is abundant information on its method, efficacy, and risk because it was commonly used as a standard agent. (2) Development of a relatively new therapeutic agent in the present circumstances is difficult. (3) Povidone-iodine is not approved as treatment in many countries, including Japan. (4) Many patients hope for conservative therapy. (5) SNI is actually performed in the real world. 

There is general agreement that the single instillation protocol is preferred because the cost associated with hospital stay can be lowered, and continuous ureteral and urethral catheterizations can be avoided [13,14,17]. However, most methods are composed of multiple instillations [2,3,8,18,19]. In contrast, we noticed that severe complications due to silver nitrate occurred in patients treated with high-concentration (≥1%) SNI [9,10,20,21,22]. In fact, there is the opinion that a high concentration (3%–5%) of silver nitrate was never used for sclerotherapy [23]. However, other investigators have suggested that efficacy is lost when sclerotherapy is performed using a low-concentration (<1%) low-volume (≤10 mL) silver nitrate solution to avoid severe complications [7]. Thus, despite a long history of this treatment, no consensus exists on the number, concentration, and volume of SNI for sclerotherapy. Based on these facts, we emphasize that discussion of a novel SNI method is not taboo. In this study, we aimed to evaluate the efficacy and safety of a novel single instillation of a low-concentration (0.2%–0.5%) high-volume (15–30 mL) protocol of SNI for patients with chyluria. 

## 2. Materials and Methods

### 2.1. Patients

Twelve patients (six men and six women) were diagnosed with chyluria between 2005 and 2018 in the Japanese Red Cross Nagasaki Genbaku Hospital. In all patients, chyluria was judged to be caused by filariasis. All patients were given conservative treatment initially, and three patients were successfully cured. Therefore, nine patients (four men and five women, a sum of 11 instillations) with chyluria were evaluated in this study. 

### 2.2. Ethics

This study was performed in accordance with the principles of the Declaration of Helsinki and the Institutional Review Board of the Japanese Red Cross Nagasaki Genbaku Hospital. Before starting this treatment, we explained to the patients that the use of SNI for chyluria is not officially allowed in Japan, and written informed consent was given by all patients. This treatment was approved by Ethic Committee at No. 559.

### 2.3. Treatment

Both cystoscopy and retrograde pyelography were performed in all nine patients. From cystoscopy, we identified the affected side based on the chylous efflux from the ureteral orifices. A 5 Fr open-ended ureteral catheter was indwelled on the affected side, and retrograde pyelography was performed using contrast media to assess the capacity and form of the renal pelvis to help decide the required instillation volume. In retrograde pyelography, pyelolymphatic fistulas were observed in some patients. Intrapelvic instillation using freshly autoclaved silver nitrate (0.1%–0.5%) (WAKO, Tokyo, Japan) was performed gently through the ureteral catheter, with about 1 min of speed adjusted to cause a mild hydronephrosis without pyelovenous backflow and peripelvic extravasation. The concentration of the used solution was judged by individual urologists (K.M., Y.I., and T.T.) based on the anesthesia method, chyluria grade, and patients’ background. However, the volume of the silver nitrate solution was judged by one urologist (T.T.) during the treatment, and all SNIs were performed under his control. If patients experienced pain and discomfort in the abdomen or back, SNI was stopped. Following sclerotherapy, ureteral catheters were immediately removed.

### 2.4. Evaluation

We examined patient characteristics, anesthesia, SNI procedure (number, concentration, and volume of injections), treatment efficacy, complications, and length of hospital stay. In addition, blood and urine tests were conducted pre- and post-treatment. We measured serum albumin, serum total protein, and urine protein levels. Success of the therapy was defined as negative conversion of both cloudy urine and proteinuria up to the last follow-up, and recurrence was characterized as milky white urine after initial clearance.

## 3. Results

### 3.1. Characteristics of the Study Population

Table 1 lists the patients’ characteristics, SNI procedures, and outcomes. The mean patient age was 70.1 years (range: 59–85), and one patient had bilateral chyluria. In principle, patients were admitted to our hospital, and SNI was then performed under either spinal or general anesthesia (seven patients and seven instillations). However, in three patients with four instillations, the therapy was performed without anesthesia because of the patient’s wishes or poor general conditions. 

### 3.2. Efficacy

In all patients, the initial treatment was 15–30 mL of 0.1%–0.5% silver nitrate solution (Table 1), and proteinuria and milky urine disappeared immediately after the end of the initial treatment. However, two patients (cases 1 and 4) had recurrence on the same side in 5 days and 4 months following the therapy, respectively. We found that recurrent cases were initially treated with a very low concentration of silver nitrate solution (0.1%). Therefore, these two patients were treated again with increased silver nitrate concentrations (0.2% and 0.5%, respectively). Both recurrent patients achieved complete symptomatic relief without recurrence for more than 6 years. Thus, the success rate by single instillation was 77.8% (7/9 patients), and the treatment in the other two patients was judged to be a success by the second course of SNI. At present, the mean, median, and range of follow-up periods after the final instillation were 73.7, 58, and 12–165 months, respectively, and all patients showed no recurrence. The mean duration of hospital stay was 6.8 days (range: 3–14). One patient (case 1) developed recurrence in 5 days, leading to prolonged hospital stay.

Table 2 shows the changes in serum albumin, serum total protein, and urine protein levels before and after treatment; complications; and duration of hospital stay. SNI improved the levels of both serum albumin and serum total protein in seven patients. As an illustration, in case 3, serum albumin and serum total protein levels before treatment decreased to 3.1 and 4.9 g/dL and improved to 4.4 and 6.8 g/dL, respectively, 6 months after the SNI treatment of chyluria.

### 3.3. Complications

Mild complications were observed in four patients. In short, three patients had flank pain (grades 1 and 2), and two experienced low-grade fever (grade 1). A single occurrence of hypotension (grade 1) and nausea (grade 1) was recorded. Hypotension in case 3 could be a side effect of spinal anesthesia, as it occurred during the operation. None of the patients experienced any severe complications. 

## 4. Discussion

In this study, we investigated the efficacy and safety of a new sclerotherapy using SNI in patients with chyluria. In the discussion of conservative treatment strategies of chyluria, we want to emphasize that the consensus regarding proper agent, dose, and number for sclerotherapy is still not established, although povidone-iodine is suggested as the most useful agent [1]. However, in the real world, silver nitrate is commonly used for sclerotherapy, even now. In fact, in 2017, a prospective study on endoscopic sclerotherapy that used silver nitrate in filarial chyluria showed that the overall success rate of silver nitrate (88.5%) is comparable to that of povidone-iodine (84.5%; *p* = 0.162) [24]. Moreover, we must be aware that povidone-iodine is not always officially allowed for the treatment of chyluria in many countries, including Japan. Actually, for both silver nitrate and povidone-iodine, the surgeon should be very careful in giving informed consent and obtaining approval from the ethical committee in these countries. Furthermore, in previous studies, cases with a relatively high risk of recurrence after sclerotherapy used povidone-iodine. In short, recurrence rates after the first course of 1% silver nitrate and 0.2% povidone-iodine instillation are 21% and 22%, respectively, and the cumulative success rates after two courses are 82% and 83%, respectively [2]. In addition, the same study showed that disease-free durations of silver nitrate and povidone-iodine were relatively short (23.6 and 20.1 months, respectively; *p* = 0.7906) [2]. Thus, information on a promising treatment protocol of sclerotherapy is essential to improve the outcome in patients with chyluria.

Our results demonstrated that seven of nine patients with chyluria (77.8%) obtained success by a single instillation of low-concentration (0.1%–0.5%) high-volume (15–30 mL) silver nitrate solution. In contrast, although two patients failed with initial instillation, they responded to the second course of SNI. Finally, all patients had no recurrence, with a mean follow-up of 69 months, and eight of nine patients had no recurrence for over 3 years. With regard to the success rate in the first course of SNI, a report revealed that 44 of 55 patients (80.0%) showed complete remission after the initial instillation of 0.5% silver nitrate, and 11 of 44 responders (25.0%) had recurrence during follow-up periods [7]. Briefly, in this report, the success rate of single SNI was 60% (33 of 55 patients). Another study showed recurrence in 11 of 44 patients after the first course of instillation in a single use of 1% silver nitrate [2]. Thus, the recurrence rate after the first course of our method appears to not be different from those in other previous reports. On the contrary, this report also showed the success rate of the second course of SNI at 62.5% (five of eight patients) [2]. As shown in Table 2, our two patients who received the second course of SNI had no recurrence during 165 and 87 months. 

Moreover, the methods and results of SNI for chyluria in previous reports are shown in Table 3 [2,3,7,8,18,19,25]. These studies used 0.1%–1.0% concentrations of silver nitrate with a small volume (<10 mL), and the success rate was 54%–75%, except in one study with good results (95%) [3]. The efficacy of our method is comparable to these results. In contrast, we noticed that over 65% of the overall success rate was obtained in methods with 3–12 courses [2,3,8,19]. Alternatively, several reports showed that the efficacy of these methods for chyluria is insufficient (60% or less), even with multiple instillations [7,18,25]. Our method, based on a single instillation, can reduce the duration of hospitalization and catheterization, costs of medical care, and the decreasing quality of life caused by long periods of catheterization. Thus, our method has various advantages compared with previous methods with multiple instillations. Table 3 shows a comparative analysis of previous studies [2,3,7,8,18,19,25]. 

As low-volume (<10 mL) and low-concentration (<1%) SNI did not always lead to a complete response and appeared to be insufficient quantitatively, multiple instillations were necessary. Even with multiple instillations, the success rates were 54%–75%, except in one study that showed good results and a 95% success rate. Although no severe complications were observed in studies with multiple instillations, the treatment regimens required longer duration of catheterization and hospitalization (at least a few days) and incurred higher medical costs compared with those in the present study.

The most important characteristic of our method is that the injected volume of silver nitrate is 15–30 mL. In general, the suitable volume of SNI is suggested to be <10 mL based on the volume of the normal renal pelvis [1]. In fact, in all methods in previous studies, the injected volume was ≤10 mL (Table 3). However, we noticed that almost all patients in our study population had a history of hydronephrosis/hydroureter and/or flank pain due to ureteral clots from the diagnosis to treatment. Moreover, imaging examinations in several previous reports showed that some patients with chyluria had hydronephrosis and flank pain resembling ureteric colic [7,8]. In short, we surmised that a solution volume of <10 mL is not insufficient to fulfil the pelvicalyceal system in patients who had a history of hydronephrosis/hydroureter, even if it was not detected by imaging examinations at the diagnosis. In fact, in all our patients, the volume of the pelvicalyceal system without pyelovenous backflow and peripelvic extravasation was judged to be 15–30 mL by retrograde pyelography. From these facts, we emphasize that a suitable amount of sclerosing solution should be decided according to the capacity of the pelvicalyceal system in each patient, and another investigator supported this opinion [26]. In contrast, many urologists believe that the instillation volume of silver nitrate must be ≤10 mL, and evidence of the efficacy and safety of SNI of >10 mL is lacking. We also agree with this opinion; however, we think that there is no evidence that an SNI of >10 mL has a significant risk. Needless to say, we suggest that an SNI of >10 mL should be strictly controlled by symptoms, findings of pyelography, and vital signs under the emergency response system.

As shown in Table 4, severe life-threatening complications have occurred with high concentrations (≥2%) of silver nitrate treatment [20,21,22]. 

In one fatal case, acute tubular necrosis developed after SNI treatment using 3.0% silver nitrate solution [21]. Another fatal case of acute hepatic and renal failure after SNI was reported; however, treatment details were not available because the patient was transferred from another hospital [11]. Finally, there is general agreement that the use of higher concentrations (>1%) of silver nitrate solution leads to a higher risk of severe complications [27]. In contrast, a case report showed that arterial hemorrhage occurred in a 30-year-old man who had a history of chyluria for 8 years and was being treated with 1.0% SNI [9]. The author commented that the cause of this condition was likely renal damage due to overinstillation and incorrect concentration. On the contrary, we noticed that severe or fatal complications did not occur in patients who were treated with <1% silver nitrate solution. From these facts, we speculated that severe complications may depend on silver nitrate concentration, and the upper limitation of the silver nitrate concentration is judged to be 0.5% in our protocol. In this discussion, we emphasize that severe adverse events were mainly dependent on SNI concentration but not on its volume. In fact, as shown in Table 4, Abraham et al. reported that 3 mL of 2% silver nitrate leads to acute renal failure [22].

This study has some limitations. First, this study has a small number of patients because filariasis is rare in Japan. In addition, our study population does not include patients aged 50 years or younger because the number of patients with filariasis has decreased remarkably since the 1970s. Second, we have no data on the changes in serum levels of triglyceride-, inflammation-, and anemia-related biochemical parameters by our method. From these facts, we emphasize that further studies with a larger study population including patients aged 50 years or younger and more detailed biochemical data are necessary to discuss the clinical usefulness and safety of our method. Third, the concentration and volume of silver nitrate solution are not unified. Therefore, further detailed prospective studies are necessary to confirm the potential efficacy and discuss the optimal protocol of SNI in patients with chyluria. Moreover, clinical trials on the efficacy and safety of second-line sclerotherapy that use low-concentration high-volume SNI for povidone-iodine-resistant chyluria are important to improve the outcome of these patients. Fourth, the efficacy and safety of our method for chyluria caused by surgery and malignancy are unclear. In planning such new treatment strategies, our results will provide crucial information. In short, high-volume (>10 mL) SNI must not be excluded if the concentration is <0.5%. On the contrary, as a future direction, information on the relationship between the pressure in the renal pelvis and usefulness or adverse events is important for the development of our method. Although conducting randomized clinical trials between povidone-iodine-based therapies and our method is extremely difficult, such studies are necessary to conclude its effectiveness in patients with chyluria.

## 5. Conclusions

Our preliminary study demonstrated that a new single instillation protocol of SNI composed of low-concentration (0.1%–0.5%) and high-volume (15–30 mL) SNI is effective and safe in patients with chyluria. Especially, the fact that most patients maintained success for over 5 years by one or two SNIs is favorably impressive. In the real world, sclerotherapies using povidone-iodine and/or silver nitrate are major tools of conservative therapy for chyluria at present. As the number of patients with chyluria will increase due to global warming and the increasing number of travelers, we would like to emphasize the importance of further, more detailed clinical trials in these patients. Even if the number of patients in this study is small, we believe that our results are important for such discussions. In addition, we suggest that a single instillation of low-concentration (0.1%–0.5%) and high-volume (15–30 mL) silver nitrate solution is a useful tool when planning new treatment strategies for chyluria. 

## Figures and Tables

**Table 1:** Patient characteristics and contents of silver nitrate instillation therapy.

Case	Sex	Age	Anesthesia	Side	Concentration (%)	Quantity (ml)
1-1	Male	79	spinal	bilateral	0.1	30, 30
1-2			no anesthesia	bilateral	0.2	25, 25
2	Female	66	spinal	left	0.5	15
3	Male	76	spinal	left	0.5	20
4-1	Female	72	no anesthesia	left	0.1	15
4-2			no anesthesia	left	0.5	25
5	Male	59	general	right	0.5	30
6	M	69	spinal	left	0.5	15
7	F	59	no anesthesia	right	0.25	15
8	F	66	spinal	left	0.5	15
9	F	85	spinal	left	0.5	30

**Table 2:** Therapeutic effect, complications, and duration of hospital stay.

Case	Therapeutic Effect					Complications	Hospital Stay (day)	Follow-up Periods (mos)
	Chyluria	Urine Protein		Serum TP (g/dL)/Alb (g/dL)				
		Pre-Tx	Post-Tx	Pre-Tx	Post-Tx			
**1-1**	**recurrence** **(5 days)**	**3+**	**±**	4.8 /2.3	5.8 /2.8	none	14	165
**1-2**	**disappearance**	±	−					
**2**	**disappearance**	3+	−	6.0 /3.7	6.6 /3.8	none	5	137
**3**	**disappearance**	4+	−	4.9 /3.1	6.8 /4.4	fever (grade 1) nausea (grade 1) hypotension (grade 1)	9	60
**4-1**	**recurrence** **(4 months)**	4+	−	5.4 /3.2	5.7 /3.4	none	3	87
**4-2**	**disappearance**	4+	−			fever (grade 1) pain (grade 1)	4	
**5**	**disappearance**	1+	−	−	−	none	9	58
**6**	**disappearance**	1+	−	−	−	pain (grade 2)	10	55
**7**	**disappearance**	3+	−	6.4 /4.0	6.9 /4.1	pain (grade 2)	6	45
**8**	**disappearance**	3+	−	6.4 /3.9	6.7 /4.0	none	4	44
**9**	**disappearance**	3+	−	5.3 /3.2	6.0 /3.6	none	4	12

Tx—treatment; mos—months.

**Table 3:** Literature review of silver nitrate instillation for the treatment of chyluria.

Authors (References)	No. Pts	Number of SNIs	Concentration (%)	Quantity (mL)	Severe Complications	Success Rate (%)
Goel et al. [2]	44	9	1.0	6–10	none	75
Dhabalia et al. [3]	59	3 or 9	1.0	5–7	none	95
Tan et al. [7]	55	1–3	0.5	10	unknown	60
Sabnis et al. [8]	62	4–12	1.0	10	none	66
Dalela et al. [25]	54	1–4	1.0	5–10	unknown	54
Dalela et al. [19]	47	3 or 9	1.0	8–10	none	68
Okamoto and Ohi [18]	129	2–10	0.1–0.5	10	unknown	59

Pts—patients; SNI—sliver nitrate instillation.

**Table 4:** List of cases with severe life-threatening complications.

Authors (References)	Number of SNIs	Silver nitrate solution		Severe Complications
		Concentration (%)	Quantity (mL)	
**Srivastava et al. [9]**	2	1	unknown	arterial hemorrhage
**Su et al. [10]**	1	1	10	acute necrotizing ureteritis
**Kulkarni et al. [11]**	1	unknown	unknown	death (acute hepatic and renal failure)
**Dash et al. [20]**	1	3	unknown	acute renal failure
**Mandhani et al. [21]**	1	3	unknown	death (acute tubular necrosis)
**Abraham et al. [22]**	1	2	3	acute renal failure

Pts—patients; SNI—sliver nitrate instillation.
